# Extracellular Matrix-Mediated Breast Cancer Cells Morphological Alterations, Invasiveness, and Microvesicles/Exosomes Release

**DOI:** 10.3390/cells9092031

**Published:** 2020-09-04

**Authors:** Marco Franchi, Zoi Piperigkou, Konstantinos-Athanasios Karamanos, Leonardo Franchi, Valentina Masola

**Affiliations:** 1 Department for Life Quality Study, University of Bologna, 47921 Rimini, Italy; 2 Biochemistry, Biochemical Analysis & Matrix Pathobiology Research Group, Laboratory of Biochemistry, Department of Chemistry, University of Patras, 26504 Patras, Greece; zoipip@upatras.gr; 3 Department of Pharmacy and Industrial Pharmacy, University of Bologna, 40100 Bologna, Italy; karamanos.akis@gmail.com; 4 Department of Medicine, University of Bologna, 40100 Bologna, Italy; leonardo.franchi@studio.unibo.it; 5 Department of Biomedical Sciences, University of Padova, 35129 Padova, Italy; valentina.masola@unipd.it; 6 Renal Unit, Department of Medicine, University Hospital of Verona, 37100 Verona, Italy

**Keywords:** breast cancer, 3D cultures, collagen, cell morphology, scanning electron microscopy (SEM)

## Abstract

Breast cancer is a leading disease in women. Several studies are focused to evaluate the critical role of extracellular matrix (ECM) in various biochemical and molecular aspects but also in terms of its effect on cancer cell morphology and therefore on cancer cell invasion and metastatic potential. ECM fibrillar components, such as collagen and fibronectin, affect cell behavior and properties of mammary cancer cells. The aim of this study was to investigate using the scanning electron microscopy (SEM) how the highly invasive MDA-MB-231 breast cancer cells, interplaying with ECM substrates during cell migration/invasion, modify their morphological characteristics and cytoplasmic processes in relation to their invasive potential. In particular we reproduced and analyzed how natural structural barriers to cancer cell invasion, such as the basement membrane (Matrigel) and fibrillar components of dermis (fibronectin as well as the different concentrations/array of type I collagen), could induce morphological changes in 3D cultures. Interestingly, we demonstrate that, even with different effects, all collagen concentrations/arrays lead to morphological alterations of breast cancer cells. Intriguingly, the elongated mesenchymal shaped cells were more prominent in 3D cultures with a dense and thick substrate (thick Matrigel, high concentrated collagen network, and densely packed collagen fibers), even though cells with different shape produced and released microvesicles and exosomes as well. It is therefore evident that the peri-tumoral collagen network may act not only as a barrier but also as a dynamic scaffold which stimulates the morphological changes of cancer cells, and modulates tumor development and metastatic potential in breast cancer.

## 1. Introduction

Tumors are characterized by a loss of tissue organization with abnormal and uncontrolled behavior of cells that grow independently. Cancer cells interact with the surrounding tissues by inducing extracellular matrix (ECM) changes similar to those found in wounds that never heal [1,2,3,4]. In solid tumors the continuous expansion of the tumor mass exerts forces on the surrounding tissues so that cancer cells lose their adhesion with neighboring cells, spread out by invading and disseminating into the surrounding microenvironment and initiate the colonization process and metastasis [5,6,7]. 

As most of cancer patient deaths are caused not by the primary tumor, but by distant metastasis, it is very important to understand why and how cancer cells gain motility and become migratory in order to penetrate into blood and lymphatic vessels and then colonize distant organs [5]. At the time cancer cells lose their cell–cell junctions and develop a migrating capability they become able to cross natural barriers like the basement membrane, thus differentiating into dangerous invasive cells through the epithelial-to-mesenchymal transition (EMT) process [8,9,10]. Cells involved in EMT process display a spindle-like or mesenchymal shape, loss of cell adhesion, inhibition of E-cadherin expression, and increased cell mobility [11,12]. 

Changes in tumor microenvironment play a critical role in tumor development and progression as well in drug efficacy [6,13,14,15]. ECM is the main component of connective tissues and includes (a) fibrillar protein constituents (collagen and elastin) transmitting and mainly resisting tensional forces, and (b) hydrophilic and water-soluble components of the ground substance (glycosaminoglycans and proteoglycans) playing an important role in buffering and hydration and opposing compressive forces [4,16,17]. ECM represents a functional and dynamic physical scaffold, able to both adapt to deformations caused by internal and external mechanical stress and selectively control the diffusion of oxygen and nutrients. Moreover, ECM plays a role in modulating the resistance that moving cells meet while crossing the collagen network of connective tissues [18,19]. 

The main component of ECM is fibrillar type I collagen that alone constitutes up to 90% protein composition of connective tissues [16,20]. Cancer cells influence peri-tumoral collagen formation but on the other hand the mechanical properties of collagen and cellular microenvironment have a great influence on cancer cell behavior [21]. In cancer progression, compressive mechanical forces resulting from tumor growth can promote invasive phenotype and cell migration. At the same time, they contribute to hypoxia through the collapse of lymphatics or small-blood vessels and the increase of interstitial fluid pressure [15,22,23]. Tumor mass rigidity or stiffness of the tumor microenvironment is largely due to increased deposition and new arrangement of ECM proteins vs. surrounding healthy tissues [24,25]. When tumors grow, ECM stiffening critically enhances the risk of metastasis [26,27,28,29,30]. This seems to be related both to the deposition of fibronectin, proteoglycans, types I, III, IV collagens, and the increase of matrix cross-linking [31,32]. 

The architecture and collagen fiber orientation of peri-tumoral stroma also modulate cancer cell migration and seem to be related to cancer progression [33,34,35,36]. In general, tumors with high invasive potential develop stiff ECMs [6], but it is also true that the organization of the collagen fibers is fundamental for exerting collagen effects on tumor cell behavior. At relatively early stages of breast cancer growth, when collagen fibers surround the tumor in a parallel bundled array, collagen acts as a dense functional barrier opposing cell invasion. However, when tumor mass expands, pressure on collagen fibers increases and both cancer and recruited associated stromal cells like cancer associated fibroblasts (CAFs) and tumor associated macrophages (TAMs) respond by adhering to fibers through integrin bindings and increasing and deforming collagen array. 

It is worth noticing that collagen interacts with tumor development by enhancing exosome secretion [37]. Exosomes are membrane-enclosed structures shed by cancer cells to communicate with other cells and interplay with ECM, but they may play a dramatic role by controlling cancer cell survival, growth, tissue invasion, and metastasis [38]. They induce the formation of CAFs in peri-tumoral stroma, favor cancer cell EMT [39], and improve the secretion of matrix metalloproteinase 14 (MMP-14) to digest and modify collagen network [40]. Crossing of macromolecules and invasive cells through the basement membrane can be facilitated by mechanical forces transmitted by peri-tumoral collagen fibers and able to alter the viscosity and permeability of the basement membrane [41]. The most dramatic peri-tumoral fiber degradation and disruption seem to occur when matrix metalloproteinases (MMPs) are released by cancer cells and penetrate among collagen fibers [42,43]. Stromal structural remodeling which strongly favors cancer cell invasion involves collagen fibers aligned perpendicularly to the tumor boundary. This collagen array and the proteolytic effect of MMPs on collagen-proteoglycan cross-links creates inter fibers/fibrils spaces where cancer cells find straight ways to migrate into the tissue [44,45,46,47]. Additionally, it was reported that recruited fibroblasts of peri-tumoral ECM can deposit both aligned fibrillar collagen and fibronectin [48,49], and their ability to produce aligned matrices is associated with the expression of the cell-surface proteoglycan syndecan-1 [35,48]. 

Different ECM proteins such as hyaluronan, laminin, MMPs, and fibronectin interact with collagen and affect cancer cell phenotype [21,49,50,51]. Both aligned type I collagen and fibronectin are largely produced by CAFs during ECM remodeling in tumor progression [48,49,52]. Together with the increased collagen cross linking, the deposition of fibronectin further contributes to enhance ECM density, cell-matrix adhesions [31,32,53,54], and cancer tumor progression. 

All data previously reported motivated us to investigate using the scanning electron microscopy (SEM) how the aggressive MDA-MB-231 breast cancer cells, when interacting with major ECM components during cell migration and invasion, alter cell morphology and cytoplasmic processes in relation to their invasive potential or capability. In particular we analyzed how structural natural barriers usually opposing cancer cell invasion, such as the basement membrane (Matrigel) and the fibrillar components of dermis (fibronectin and different concentrations and array of type I collagen fibrils) could affect the morphology and invasion capability of MDA-MB-231 breast cancer cells.

## 2. Materials and Methods

### 2.1. Cell Cultures

MDA-MB-231 breast cancer cells (obtained from the American Type Culture Collection (ATCC) were cultured in DMEM-High Glucose (EuroClone, Pero (MI), Italy.) (17.5 mM glucose) supplemented with 10% fetal bovine serum (FBS) (Biochrome AG, Merck KGaA, Darmstadt, Germany), L-glutamine (2 mM), penicillin (100 U/mL), and streptomycin (100 µg/mL), at 37 °C in a humidified atmosphere with 5% CO_2_. Cells were checked for the absence of mycoplasma by Venor™ GeM Mycoplasma Detection Kit (Sigma-Aldrich, Merck KGaA, Darmstadt, Germany). Cells at 80% of confluences were detached with Trypsin-EDTA solution and were seeded on four polystyrene flasks for 2-D control cultures, on four “Isopore Membrane Filters” with a pore size of 8.0 µm (Millipore, Milan, Italy) for 3-D control cultures. To mimic structural ECM natural barriers opposing to cancer cell invasion (2.5 × 10^5^) cells were seeded for 4 h on 20 similar filters coated with fibronectin (130 µg/mL, BD Becton Dickinson, East Rutherford, NJ, USA) or Matrigel (3.0 µg/µL, BD Biosciences, Milan, Italy) or type I Collagen solution in different concentrations (50,200 and 3000 µg/mL C3867, Sigma-Aldrich, Schnelldorf, Germany). Fibronectin, collagen, and Matrigel were diluted at the proper concentration in sterile water (pH 6) for 2 h at 37 °C. The lower chamber was filled with DMEM-High Glucose with 20% FBS. Cells were also directly seeded on four commercial lyophilized and compressed collagen membranes of tightly packed type I collagen fibers from equine Achilles tendon (Bioteck, Milan, Italy). The lower chamber was filled with DMEM-High Glucose with 20% FBS.

### 2.2. Scanning Electron Microscopy

Breast cancer cells seeded in both 2D and 3D cultures were fixed in a Karnovsky’s solution for 30 min at 4 °C. Portions of the flasks (2D cultures) and the intact Millipore filters alone or covered by different substrates (3D cultures) with adhered cells were rinsed three times with 0.1% Sodium Cacodylate Buffer pH 7, dehydrated with increasing concentrations of ethanol, and finally dehydrated with hexamethyldisilazane (Sigma-Aldrich, Inc., St. Louis, MI, USA) for 15 min. One sample of each Millipore group was mechanically sectioned in two portions by a razorblade. All specimens were mounted on appropriate stubs, coated with a 5 nm palladium gold film (Emitech 550 sputter-coater) to be observed under a SEM (Philips 515, Eindhoven, The Netherlands) operating in secondary-electron mode.

## 3. Results

### 3.1. Breast Cancer Cell Phenotypes in 2D Cultures

**Figure 1:**
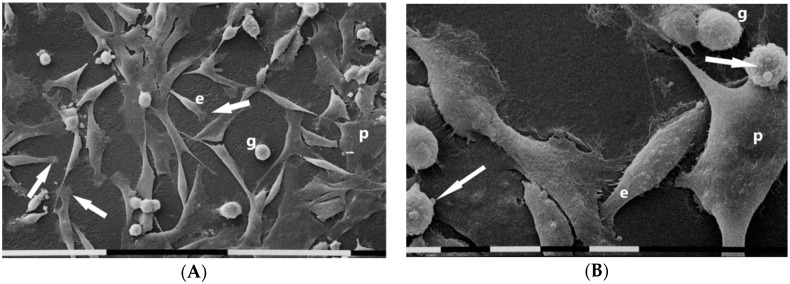
Scanning electron microscopy of MDA-MB-231 cells cultured in 2D flask cultures. Cells only partially showed cell–cell contacts and mainly included “cobblestone” shaped flattened polygonal cells (p) and “squid” shaped elongated cells (e) with lamellipodia (arrows). Only very few isolated globular cells (g) were present. White bar = 100 µm (**A**). Three different cell phenotypes were distinguishable: flattened polygonal one (p), an elongated cell (e), and globular ones (g). Sparse microvilli were present on all cell surfaces and few microvesicles (arrows) were detectable on an elongated cell and globular ones. White bar = 10 µm (**B**).

SEM analysis of MDA-MB-231 cells cultures in 2D flasks showed different and equally distributed cell phenotypes: (a) “squid” shaped or isolated and elongated cells exhibiting lamellipodia at one end, (b) “cobblestone” shaped or flattened polygonal cells only partially showing cell–cell contacts, (c) fewer isolated globular shaped cells (Figure 1A). A low amount of microvilli were detectable on flattened polygonal cell surface whereas few microvesicles were observed on the cytoplasmic surface of both globular and elongated ones (Figure 1B).

### 3.2. Breast Cancer Cells Phenotypes in 3D Cultures on Millipore 

**Figure 2:**
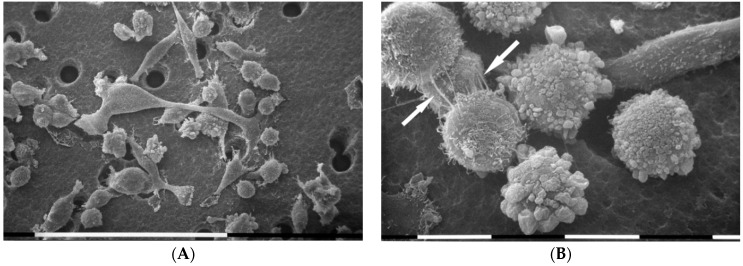
Scanning electron microscopy of MDA-MB-231 cells cultured in Millipore filter. Two main cell phenotypes were observed: both globular cells and squid elongated ones showing lamellipodia and migrating through the Millipore filter holes. Microvilli and microvesicles mainly covering the globular cells were visible. White bar = 100 µm (**A**). Some globular cells show microvilli and cytoplasmic intercellular connections or tunneling nanotubes (TNTs) (arrows). Other globular cells exhibit many microvesicles. On the right and upside, an elongated cell showing only microvilli appeared to cross a Millipore filter hole. White bar = 10 µm (**B**).

It is worth noticing that when MDA-MB-231 cells were cultivated on Millipore filter the flattened polygonal phenotype strongly decreased as compared to non-Millipore control cultures. On the other hand, the elongated cells with polarized lamellipodia and the globular ones were still detectable. A number of cells of all phenotypes were observed to fill and migrate through the Millipore holes (Figure 2A). All cells showed microvilli and microvesicles on their surfaces. However, microvilli were mostly located on flattened polygonal cells. A number of globular cells were covered by microvilli, whereas others were completely covered only by microvesicles of different sizes. Straight and very thin intercellular cytoplasmic protrusions appeared suspended from the culture ground and occasionally connected with adjacent cells. As shown in Figure 2B, these thin intercellular connections (50–300 nm in diameter) displaying a cylindrical shape corresponded to previously described tubular tunneling nanotubes (TNTs) [55,56,57].

### 3.3. Breast Cancer Cells on Fibronectin-Covered Millipore 

**Figure 3:**
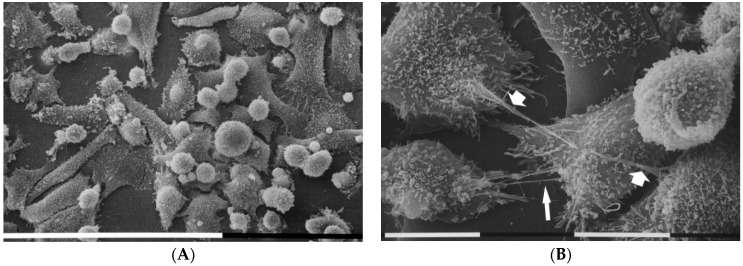
Analysis of cell morphology in 3D cultures on Millipore filter covered by fibronectin (130 µg/mL) by scanning electron microscope. MDA-MB-231 cells cultured on fibronectin showed many “cobblestone” flattened polygonal cells and globular shaped ones, but few elongated ones. All cells exhibited both microvilli and many microvesicles. White bar = 100 µm (**A**). Flattened polygonal cells showing microvilli appeared to be connected by thin single TNTs (narrow arrow) and thicker ones (wide arrows) composed by single thin TNTs tightly bundled together. On the right, a globular cell exhibited microvesicles on the cytoplasmic surface. White bar = 10 µm (**B**).

MDA-MB-231 cells cultivated in 3D cultures on Millipore filter covered by fibronectin (130 µg/mL) showed an increased cellular density probably due to a higher cell adhesion as compared to the control groups. Similarly, all cells showed more microvilli and microvesicles as compared to the same cells cultured on Millipore alone. Globular shaped cells and flattened polygonal ones were more prominent than elongated cells. Importantly, differences in the cytoplasmic protrusions were also evident, as flattened polygonal cells showed only microvilli, whereas microvesicles were observable on globular shaped cells (Figure 3A). Some adjacent cells were connected by thin single TNTs (50–300 nm in diameter) and thicker conical shaped ones (also 900 nm in diameter) corresponding to a fascicle of single thin TNTs tightly bundled together (Figure 3B).

### 3.4. Breast Cancer Cells on Matrigel-Coated Millipore 

**Figure 4:**
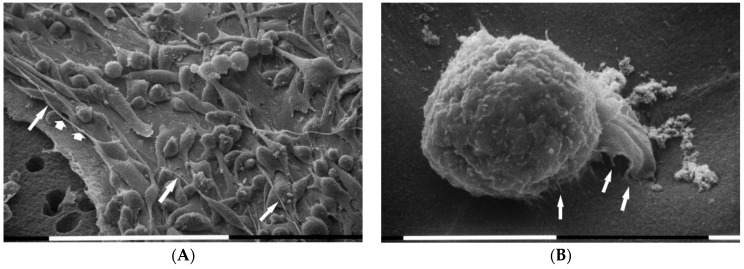
Scanning electron microscopy (SEM) micrographs of 3D cultures on Millipore filter covered by concentrated Matrigel solution (3.0 µg/µL). MDA-MB-231 cells intimately appeared to be attached to the Matrigel layer. In cases where the Millipore filter was not covered by Matrigel (bottom left side of 4A), an elongated cell has almost completely crossed a non-Matrigel coated Millipore filter hole. Three phenotypes with microvilli and microvesicles were identified: the globular shaped cells, the flattened polygonal ones, and the elongated cells. In particular, some elongated cells developing long and thin filopodia (narrow arrows) sometimes containing microvesicles (wide arrows) display an evident fusiform shape. White bar = 100 µm (**A**). Short filopodia which morphologically could also resemble developing invadopodia (arrows) and a fully developed larger invadopodia (on the right) of a globular cell during the invasion of Matrigel are shown next to the substrate surface. Exosomes and microvescicles next to Matrigel surface can be seen on the left-upside and near the globular cell. White bar = 10 µm (**B**).

It is worth noticing that when the MDA-MB-231 cells were cultured on a concentrated Matrigel layer (3.0 µg/µL) they intimately adhered to the substrate. Three cell morphologies were noted: globular shaped cells and elongated ones grown on the flattened polygonal ones. All cells exhibited both microvilli and microvesicles. Some elongated cells developing long and thin polarized filopodia at their both ends presented a clear fusiform-elongated shape (Figure 4A). Moreover, isolated cells intimately attached to the Matrigel layer and invading the surface of the substrate developed short cytoplasmic protrusions morphologically comparable to invadopodia. Although SEM analysis does not allow to observe a dynamic event like invadopodia formation, invadopodia were recognized as relatively short cytoplasmic protrusions originating from the ventral side of the cells. Notably, shed exosomes and microvescicles could also be detectable on the Matrigel surface (Figure 4B).

### 3.5. Breast Cancer Cells on Various Concentrations of Collagen-Covered Millipore 

**Figure 5:**
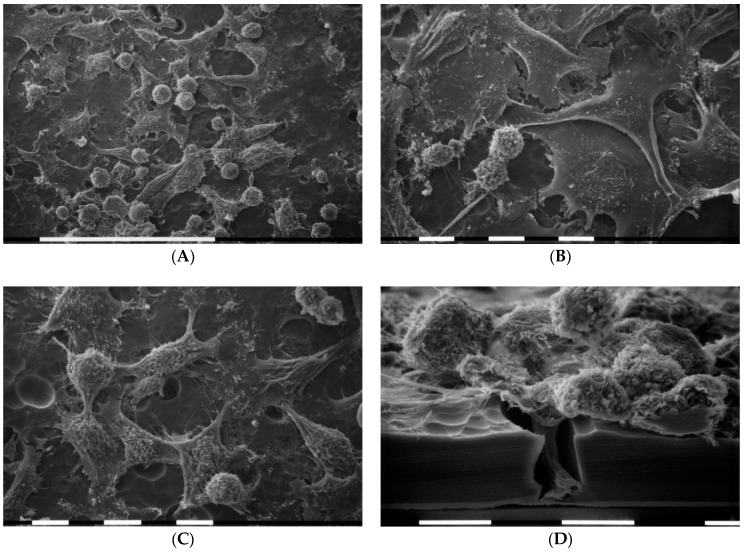
Cell morphology of MDA-MB-231 breast cancer cells grown on Millipore filter covered by type I collagen (50 µg/mL). SEM analysis of 3D cultures showed that MDA-MB-231 cells include almost equally distributed and relatively isolated cells showing elongated, globular, and flattened polygonal shapes. Millipore filter was not completely covered by collagen so that its rough surface was discernable. White bar = 100 µm (**A**). Cells showed microvilli and microvesicles on their surface. Flattened polygonal cells mainly exhibited few superficial microvilli and showed some cell–cell contact (**B**), whereas microvesicles were mostly distributed on globular and elongated cells which display short and thin filopodia radially spread from the cells. White bar = 10 µm (**B**,**C**). Lateral view of a razorblade sectioned Millipore filter. Observe the wave rough porous Millipore surface: a collective invasion with a group of cells is taking place. The leader cell shows a “funnel” shape while invaginates and crosses a Millipore hole. White bar = 10 µm (**D**).

In order to evaluate the importance of collagen type I on the morphology of breast cancer cells and particularly how the different concentrations that form a series of arrays on Millipore filter may be critical factors for the cell functional properties and invasiveness, three different concentrations of collagen type I (50,200 and 3000 µg/mL) were utilized. As starting point the lowest concentration was used. Although most diluted collagen solution (50 µg/mL) did not completely cover the Millipore filter, which showed an exposed rough surface, most of the Millipore holes were partially filled by intertwined collagen fibrils. When MDA-MB-231 cells grew on the Millipore filter covered by diluted type I collagen they included equally distributed and relatively isolated cells with elongated, globular, and flattened polygonal shapes (Figure 5A). Flattened polygonal cells mainly showed few superficial microvilli and cell–cell contact, whereas microvesicles were mostly distributed on globular and elongated ones. These two phenotypes displayed short and thin filopodia radially spreading from the cells and intertwined to each other (Figure 5B,C). Cell migration was confirmed by both individual and collective invasion with the leader cell assuming a “funnel” shape while crossing the Millipore holes (Figure 5D).

**Figure 6:**
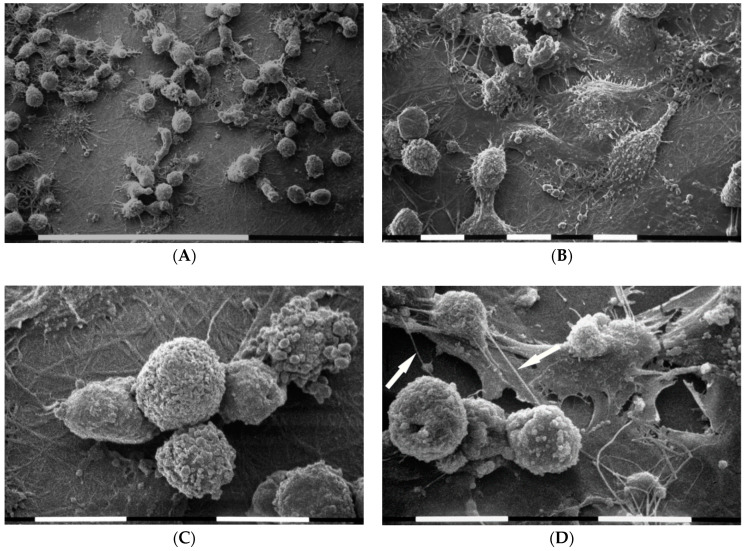
MDA-MB-231 cells in 3D cultures on Millipore filter covered by type I collagen (200 µg/mL). Most of cells correspond to isolated globular cells which showed many thin filopodia attaching to the collagen fibrils of the substrate and radially spreading from each cell. White bar = 100 µm (**A**). At a larger magnification all cell microvilli and microvesicles (**B**). Microvesicles distributed on the surface of adjacent globular cells showed different sizes. Collagen fibrils strongly adhering on the Millipore surface were detectable on the ground. White bar = 10 µm (**C**). Thin intercellular TNTs (arrows) connect adjacent globular cells grown on flattened polygonal ones. White bar = 10 µm (**D**).

MDA-MB-231 cells growing on a layer of higher type I collagen concentration (200 µg/mL) were mostly composed of isolated globular cells which developed many thin filopodia attaching to the collagen fibrils and radially spreading from each cell (Figure 6A). All cells showed microvilli and microvesicles on their cytoplasmic surface, but microvesicles which exhibited different sizes were much more distributed on the globular cells surface (Figure 6B,C). Thin and straight cytoplasmic intercellular protrusions corresponding to intercellular TNTs connected adjacent cells (Figure 6D).

**Figure 7:**
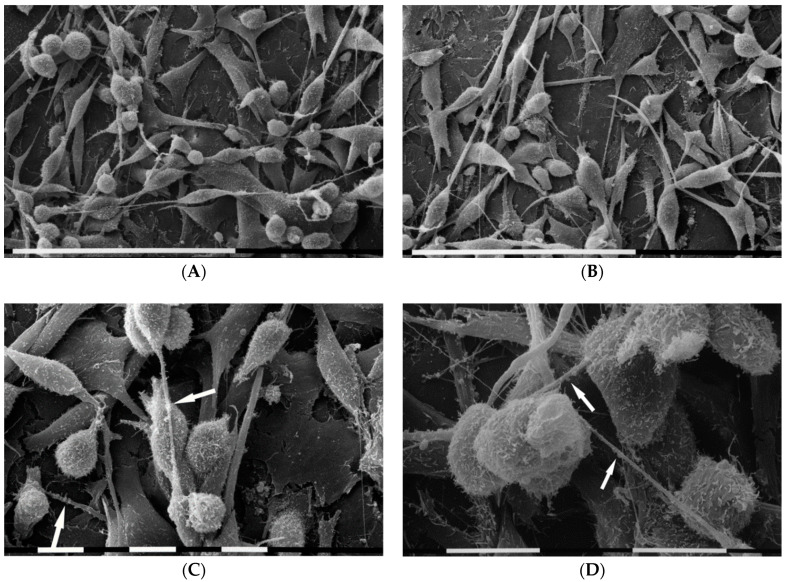
MDA-MB-231 cells in 3D cultures on Millipore filter covered by type I collagen (3000 µg/mL). Breast cancer cells grew in more than one layer on the highest concentration of type I collagen substrate, but no tight cell–cell contact was visible. Only the flattened polygonal cells appeared directly in contact with the collagen fibrils, whereas both elongated cells showing a fusiform shape and globular ones grow over the flattened ones. The three different phenotypes were equally distributed, and all cells showed microvilli and microvesicles. Long and thin filopodia originating from elongated cells, thus appearing like fusiform shaped, and TNTs were apparently distinguishable. White bar = 100 µm (**A**,**B**). Some long filopodia exhibited exosomes (not easily visible) and microvesicles (arrows) on their surface. White bar = 10 µm (**C**,**D**).

When MDA-MB-231 cells grew at the highest concentration of type I collagen fibrils (3000 µg/mL) the three previously described different phenotypes were almost equally represented and all cells showed both microvilli and microvesicles. A very high cell density was observed with cells arranged in sandwich layers: the flattened polygonal cells were directly attached to the collagen fibrils, whereas both the elongated cells exhibiting a more definite fusiform shape and the globular ones grew over, but showed no cell–cell contacts. In this group many long and thin filopodia mostly originating from the elongated cells and similar to those described in concentrated Matrigel substrate were developed. Additionally, intercellular TNTs were much more observable as compared with the samples of all previous groups (Figure 7A,B). A number of thin and long filopodia exhibited exosomes and microvesicles on their surface (Figure 7C,D).

**Figure 8:**
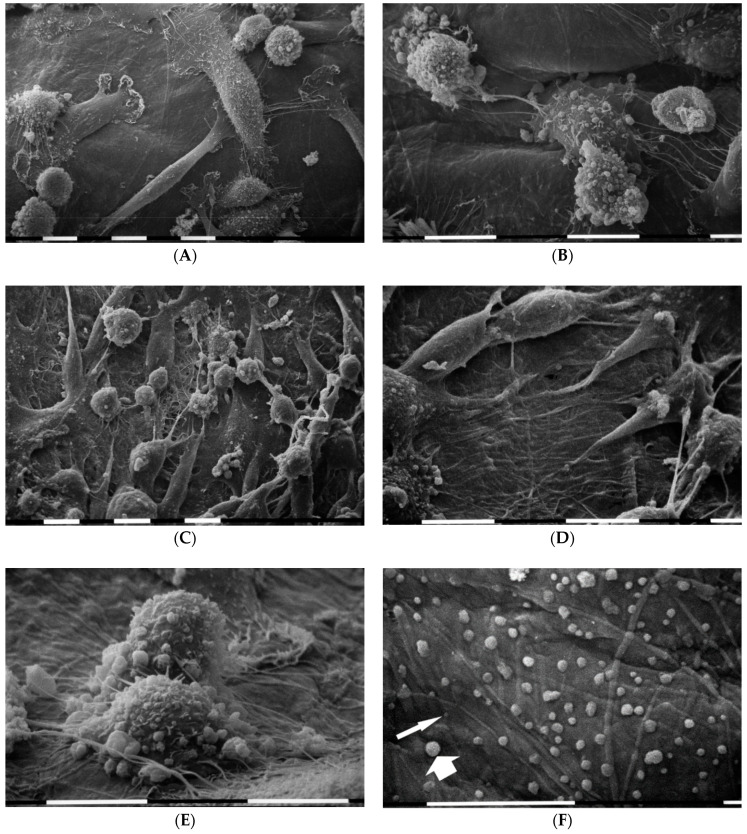
3D cultures on a membrane of densely packed collagen fibers. MDA-MB-231 cells showing microvilli and microvesicles on the cytoplasmic surface appeared to be attached to the dense collagen fiber membrane and included few isolated flattened polygonal cells, globular and polarized “squid” elongated ones which show lamellipodia. Thin intercellular connections or TNTs were visible between adjacent cells. White bar = 10 µm (**A**,**B**). In areas where fibrils of the collagen membrane were more exposed, elongated and fusiform shaped cells appear. White bar = 10 µm (**C**,**D**). A TNT intercellular connection was also visible on the right side. White bar = 10 µm (**D**). Two globular cells exhibiting many microvesicles and developing short cytoplasmic protrusions attached to exposed collagen fibrils. The globular cell in the foreground partially invaginates into collagen substrate. White bar = 10 µm (**E**). Many larger microvesicles (wide arrow) and smaller exosomes (narrow arrow) shed by MDA-MB-231 cells are detectable on the superficial intertwined fibrils of the collagen membrane. White bar = 10 µm (**F**).

When MDA-MB-231 cancer cells were seeded on a commercial lyophilized and compressed collagen membrane of tightly packed type I collagen fibers from equine Achilles tendon, isolated cells exhibited both microvilli and microvesicles on their cytoplasmic surface. They were intimately attached to the dense collagen fibrils and included few isolated flattened polygonal cells, globular and “squid” elongated ones which showed lamellipodia. Some adjacent cells developed thin intercellular connections or TNTs (Figure 8A,B). In areas where fibrils of the collagen membrane appeared more exposed and recognizable, elongated and fusiform shaped cells displaying filopodia were more frequently observed (Figure 8C,D). In similar areas the globular cells exhibiting many microvesicles and developing short cytoplasmic protrusions in contact with the collagen fibrils seemed to invaginate (Figure 8E). Many microvesicles and exosomes shed by MDA-MB-231 cells were recognizable on the superficial intertwined fibrils of the collagen membrane (Figure 8F).

## 4. Discussion

When a tumor mass grows, mechanical forces are transmitted to the peri-tumoral microenvironment and induce either direct or CAFs-mediated structural changes of collagen array in ECM. On the other hand, the newly formed collagen array interacts with cancer cells by inducing migratory cell phenotypes and tumor progression. In normal and pathologic conditions, cell–ECM interactions are largely mediated via integrins, a large family of cell-surface receptors which upon transferring both mechanical and chemical signaling also control cytoskeleton array and activate intracellular signaling pathways [58,59,60]. Breast carcinoma cells in 3D collagen gel cultures generate nearly constant forces that are related to collagen concentration and matrix stiffness [61,62]. Through integrins both biochemical and biomechanical changes of ECM promote and develop tumor progression modifying the cytoskeleton of cancer cells [19,60,63]. During microenvironment invasion, changes of cancer cell cytoskeleton are strongly related to structural modifications of cancer cell phenotypes. 

Breast tumorigenesis is accompanied by new collagen crosslinking, ECM stiffening, and increased focal adhesions [59]. The morphological study of breast cancer cell phenotypes in 3D cultures with different concentrations and array of collagen fibrils allows to better understand how changes of tumor microenvironment can modify the cancer cell behavior. In 2D flask cultures MDA-MB-231 isolated cells exhibited three almost equally distributed phenotypes: the mesenchymal elongated shaped cells which are morphologically the expression of EMT and increased invasive potential, the polygonal flattened cells comparable to an epithelial phenotype, and the globular ones. It is noteworthy that cell surface showed both few microvesicles and microvilli related to the shedding of exosomes which can contain MMPs among other matrix effectors [64,65]. In 3D cultures on Millipore filter alone the same cells showed fewer flattened polygonal cells and increasing of microvilli especially on the surface of globular shaped cells vs. the 2D cultures. The reduction of the flattened epithelial-like cells, the increase of microvilli, and occasional intercellular TNTs between adjacent cells suggest that the rough 3D Millipore filter surface with relatively large holes per se may stimulate cell migration. Even though there is evidence about TNT existence, the formation mechanism and function still remain to be fully understood. However, it is interesting that TNTs are involved in both vesicles and mitochondrial intercellular transfer [66,67] and treatments exerting stressful insults seem to evoke TNTs formation [67]. 

The presence of fibronectin on Millipore filter evoked an increased cell density probably related to higher cell adhesion to the substrate but also a slight decrease of the elongated mesenchymal cells was detectable. Cell-to-cell direct communications seemed to be improved in some cells which developed single and large composed intercellular TNTs.

Epithelial basement membrane containing a thin continuous layer of collagen IV, laminin, fibronectin, and several types of proteoglycans represents the first physical barrier that migratory cancer cells have to cross when they start ECM invasion [60]. Both macromolecules and invasive cells are favored in crossing through the basement membrane when mechanical forces transmitted by peri-tumoral collagen fibers modify the viscosity and permeability of the natural connective barrier [41]. Basement membrane is usually reproduced in vitro by a gelatinous protein mixture or Matrigel [68]. We used a very high concentration of Matrigel (3.0 µg/µL) with the purpose to obtain a continuous and thick physical layer completely covering the Millipore holes. In these cultures, breast cancer cells displayed a good adhesion to the Matrigel substrate. Moreover, they showed a new phenotype: very elongated fusiform (spindle-like) shaped cells, probably originating from the mesenchymal elongated ones, exhibiting long and thin filopodia with exosomes and microvesicles on their surface. This may be attributed to the fact that Matrigel (basement membrane) barrier has an epigenetic effect on cancer cell phenotypes which turn them into mesenchymal invasive cells. Confirming the effect of Matrigel in stimulating cancer cells invasion, many exosomes and microvesicles related to an increased vesicles shedding were observed on the Matrigel surface. Moreover, some cells showed invadopodia which as reported in literature usually contain MMPs and validate the increased cell invasiveness [69]. 

Even though the interstitial ECMs mainly consist of type I and type III collagens, but also include fibronectin and proteoglycans, all collagen substrates used to cover the Millipore filters or the collagen membrane itself contained only type I collagen, the most abundant type in ECM [16,60]. Despite the fact that all groups included the same biochemical component (type I collagen) the collagen substrates showed different thicknesses and variable porosity due to the different collagen concentrations which affected also collagen fibrils array. A very diluted collagen solution (50 µg/mL) did not completely cover the Millipore filter even though intertwined collagen fibrils usually filled most of the Millipore holes. In these samples the three almost equally distributed cancer cell phenotypes observed in 2D cultures were still detectable. Even though some of the flattened polygonal cells showed few cell–cell contacts, most of cells appeared isolated and displayed microvilli, microvesicles, and short thin filopodia interacting with the sparse collagen fibrils. In mesenchymal tumors, including melanoma and fibrosarcoma, the density of the ECM seems to determine the invasion mode of mesenchymal tumor cells independently on matrix stiffness: fibrillar, high porosity ECM enables single-cell invasion, whereas dense matrix induces a collective migration as an obligatory protease-dependent process related to MMPs activity [70]. However, in the high porosity but low concentrated collagen group (50 µg/mL) both individual and collective invasion, with “funnel” shaped leader cell invaginating into Millipore holes, were observed. These data suggest that the cells became able to modify their phenotype characteristics (plasticity) as to survive and cross hostile microenvironments [19,20,60]. Notably, it has been reported that collective cell migration seems to have higher invasive capacity and resistance to clinical treatments than individual cell migration [71]. 

Differently, cultures of the MDA-MB-231 cells on Millipore filter covered by a more concentrated type I collagen (200 µg/mL) mostly included isolated globular cells which showed many thin filopodia adhering to the collagen fibrils and radially spreading from each cell. Interestingly, some filopodia showed also exosomes on their cytoplasmic surface. It has been reported that fibroblasts filopodia seem to have a direct mechanical role in exosome capture and represent highways favoring exosomes penetration into the cell [72]. However, in breast cancer cells it was suggested that they might also play sensory and mechanical roles during cell invasion [73]. The presence of microvilli and also microvesicles on cell surface still suggested an aggressive behavior of the cells. 

When breast cancer cells were cultured on Millipore covered by high concentrated (3000 µg/mL) type I collagen, cells with no tight cell–cell contact and covered by many microvilli and microvesicles grew in more than one layer. It is, therefore, plausible to suggest that these cells attaching very well to the concentrated collagen substrate and developing an intense vesicles production are preparing to invade the thick collagen layer. Additionally, the many long filopodia covered by microvesicles and exosomes and originating from fusiform cells together with many TNTs are all structural changes which may suggest that an EMT process is occurring. 

MDA-MB-231 cells growing on a commercial lyophilized and compressed collagen membrane of tightly packed type I collagen fibers from equine Achilles tendon displayed all three cancer cell phenotypes covered by many microvesicles and short filopodia, but some globular cells rich in microvesicles seemed to try to invaginate into more exposed collagen fibrils. Several microvesicles and exosomes released by cells and attached to the collagen membrane surface demonstrated an increased vesicles production/release. It was reported that collagen interacts with tumor development by enhancing exosome secretion [36]; thus, collagen and exosomes should be jointly considered as important factors promoting cancer progression [21]. Additionally, the development of intercellular TNTs which are involved in both vesicles and mitochondrial intercellular transfer [66,67] are expression of cell–cell cross talk and indicate an increased cell activity. 

In conclusion, cross talk between breast cancer cells observed in 3D Millipore cultures as compared to 2D flask cultures, is enhanced by fibronectin substrate which ensures an increased cell adhesion to the ground culture and promotes increasing vesicles production [49]. When cells encounter a thick layer of Matrigel they are stimulated to develop also invadopodia while attempting to cross the basement membrane [74]. Collagen, always favoring morphological changes, seems to interact with breast cancer of the various phenotypes in relation to the different concentrations which define different collagen fibrils arrays and porosity [70,75]. A very low collagen concentration (50 µg/mL) does not represent a valid barrier to invading cells because the wide interfibrillar spaces permit both individual and collective invasion. A more concentrated collagen substrate (200 µg/mL) induces the development of both sensing long filopodia and intercellular TNTs. However, a very impressive cell adhesion to collagen fibrils is evident in samples with the most concentrated collagen network (3000 µg/mL): cells arrange in more than one layer and mesenchymal fusiform cells with very long filopodia covered by many microvesicles and exosomes are prominent.

Finally, the commercial lyophilized and compressed collagen membrane of tightly packed type I collagen fibers from equine Achilles tendon, including randomly intertwined fibrils at the surface, does not allow MDA-MB-231 cell invasion. Nevertheless, cells exhibit fusiform (spindle-like) EMT morphological phenotype with long filopodia, intercellular TNTs, and many microvesicles and exosomes on cell surface but also shed on the collagen membrane. Even in absence of mechanical pressure loads exerted by tumor growth, breast cancer cells change their morphology into an invasive shape, thus confirming that dense collagen fibers per se physically interact with MDA-MB-231 cancer cells and stimulate their invasive potential.

Regarding the role of collagen on cancer cell morphology and aggressiveness, a number of studies are more focused on collagen stiffness rather than on collagen arrangement [27,28,31,32,34,44,59,76,77,78], whereas other studies report that collagen fibrils array and pore size have a strong influence on tumor progression [33,34,35,36,79,80,81]. In this study, we clearly demonstrated that even with a different mechanism of action all collagen parameters probably contribute to change morphological breast cancer phenotypes. Intriguingly, we showed in our experiments that the elongated fusiform shaped cells, typically exhibiting a mesenchymal phenotype as the result of an EMT, were more prominent in 3D cultures with a dense and thick substrate (Matrigel, higher concentrations of collagen and commercial lyophilized compressed collagen membrane). However, independently on their shape, all cells able to produce and release microvesicles and exosomes, that among other effectors may contain MMPs, have to be considered dangerous for collagen integrity. It is evident that ECM collagen network can regulate tumor growth by acting both as a barrier opposing cell invasion and drug penetration, but also as a scaffold which favors tumor development and metastasis through the promotion of mesenchymal phenotypes. 

Although the analysis of the effect of ECM on breast cancer cells only at the morphological level and the use of only one cell line represents a limitation of this study, our results add important insights and knowledge, and will be helpful for the comparison between forthcoming biochemical and morphological investigations to completely explain the intriguing but dramatic interplay between collagen and cancer cells.

## References

[1] Schäfer M., Werner S. (2008). Cancer as an overhealing wound: An old hypothesis revisited. Nat. Rev. Mol. Cell Biol..

[2] Fouad Y.A., Aanei C. (2017). Revisiting the hallmarks of cancer. Am. J. Cancer Res..

[3] Foster D.S., Jones R.E., Ransom R.C., Longaker M.T., Norton J.A. (2018). The evolving relationship of wound healing and tumor stroma. JCI Insight.

[4] Paolillo M., Schinelli S. (2019). Extracellular Matrix Alterations in Metastatic Processes. Int. J. Mol. Sci..

[5] Hanahan D., Weinberg R.A. (2000). The hallmarks of cancer. Cell.

[6] Nagelkerke A., Bussinkù J., Rowan A.E., Span P.N. (2015). The Mechanical Microenvironment in Cancer: How Physics Affects Tumours. Semin. Cancer Biol..

[7] Martins Cavaco A.C., Dâmaso S., Casimiro S., Costa L. Collagen biology making inroads into prognosis and treatment of cancer progression and metastasis. Cancer Metastasis Rev..

[8] Hirohashi S., Kanai Y. (2003). Cell Adhesion System and Human Cancer Morphogenesis. Cancer Sci..

[9] Gavert N., Ben-Ze’ev A. (2008). Epithelial-mesenchymal transition and the invasive potential of tumors. Trends Mol. Med..

[10] Yilmaz M., Christofori G. (2009). EMT, the Cytoskeleton, and Cancer Cell Invasion. Cancer Metastasis Rev..

[11] Pradella D., Naro C., Sette C., Ghigna C. (2017). EMT and stemness: Flexible processes tuned by alternative splicing in development and cancer progression. Mol. Cancer.

[12] Vargas D.A., Bates O., Zaman M.H. (2013). Computational Model to Probe Cellular Mechanics During Epithelial-Mesenchymal Transition. Cells Tissues Organs.

[13] Bissell M.J., Radisky D.C., Rizki A., Weaver V.M., Petersen O.W. (2002). The organizing principle: Microenvironmental influences in the normal and malignant breast. Differentiation.

[14] Correia A.L., Bissell M.J. (2012). The tumor microenvironment is a dominant force in multidrug resistance. Drug Resist. Updates.

[15] Spill F., Daniel S., Reynolds D.S., Kamm R.D., Zaman M.H. (2016). Impact of the Physical Microenvironment on Tumor Progression and Metastasis. Curr. Opin. Biotechnol..

[16] Theocharis A.D., Skandalis S.S., Gialeli C., Karamanos N.K. (2016). Extracellular matrix structure. Adv. Drug Deliv. Rev..

[17] Karamanos N.K., Piperigkou Z., Theocharis A.D., Watanabe H., Franchi M., Baud S., Brezillon S., Gotte M., Passi A., Vigetti D. (2018). Proteoglycan Chemical Diversity Drives Multifunctional Cell Regulation and Therapeutics. Chem. Rev..

[18] Eble J.A., Niland S. (2019). The extracellular matrix in tumor progression and metastasis. Clin. Exp. Metastasis.

[19] Kumar S., Weaver V.M. (2009). Mechanics, malignancy, and metastasis: The force journey of a tumor cell. Cancer Metastasis Rev..

[20] Wolf K., Alexander S., Schacht V., Coussens L.M., von Andrian U.H., van Rheenen J., Deryugina E., Friedl P. (2009). Collagen-based cell migration models in vitro and in vivo. Semin. Cell Dev. Biol..

[21] Xu S., Xu H., Wang W., Li S., Li H., Li T., Zhang W., Yu X., Liu L. (2019). The role of collagen in cancer: From bench to bedside. J. Transl. Med..

[22] Tse J.R., Engler A.J. (2011). Stiffness Gradients Mimicking In Vivo Tissue Variation Regulate Mesenchymal Stem Cell Fate. PLoS ONE.

[23] Stylianopoulos T., Martin J.D., Snuderl M., Mpekris F., Jain S.R., Jain R.K. (2013). Coevolution of Solid Stress and Interstitial Fluid Pressure in Tumors During Progression: Implications for Vascular Collapse. Cancer Res..

[24] Jonietz E. (2012). Mechanics: The forces of cancer. Nature.

[25] Garra B.S., Neu C.P., Genin G.M. (2015). Ultrasound imaging of mechanical properties of cancer. Handbook of Imaging in Biological Mechanics.

[26] Wolfe J.N. (1976). Risk for breast cancer development determined by mammographic parenchymal pattern. Cancer.

[27] Boyd N.F., Dite G.S., Stone J., Gunasekara A., English D.R., McCredie M.R., Giles G.G., Tritchler D., Chiarelli A., Yaffe M.J. (2002). Heritability of mammographic density, a risk factor for breast cancer. N. Engl. J. Med..

[28] Boyd N.F. (2013). Mammographic densities and breast cancer risk. Am. Soc. Clin. Oncol. Educ. Book.

[29] Barcus C.E., Keely P.J., Eliceiri K.W., Schuler L.A. (2013). Stiff collagen matrices increase tumorigenic rolactin signaling in breast cancer cells. J. Biol. Chem..

[30] Xiong G., Deng L., Zhu J., Rychahou P.G., Xu R. (2014). Prolyl-4-hydroxylase alpha subunit 2 promotes breast cancer progression and metastasis by regulating collagen deposition. BMC Cancer.

[31] Zhu G.G., Risteli L., Makinen M., Risteli J., Kauppila A., Stenback F. (1995). Immunohistochemical study of type I collagen and type I pN-collagen in benign and malignant ovarian neoplasms. Cancer.

[32] Oudin M.J., Jonas O., Kosciuk T., Broye L.C., Guido B.C., Wyckoff J., Riquelme D., Lamar J.M., Asokan S.B., Whittaker C. (2016). Tumor Cell-Driven Extracellular Matrix Remodeling Drives Haptotaxis during Metastatic Progression. Cancer Discov..

[33] Painter K.J. (2009). Modelling cell migration strategies in the extracellular matrix. J. Math. Biol..

[34] Egeblad M., Rasch M.G., Weaver V.M. (2010). Dynamic interplay between the collagen scaffold and tumor evolution. Curr. Opin. Cell Biol..

[35] Riching K.M., Cox B.L., Salick M.R., Pehlke C., Riching A.S., Ponik S.M., Bass B.R., Crone W.C., Jiang Y., Weaver A.M. (2014). 3D collagen alignment limits protrusions to enhance breast cancer cell persistence. Biophys. J..

[36] Brassart-Pasco S., Brézillon S., Brassart B., Ramont L., Oudart J.B., Monboisse J.C. (2020). Tumor Microenvironment: Extracellular Matrix Alterations Influence Tumor Progression. Front. Oncol..

[37] Szvicsek Z., Oszvald A., Szabo L., Sandor G.O., Kelemen A., Soos A.A., Paloczi K., Harsanyi L., Tolgyes T., Dede K. (2019). Extracellular vesicle release from intestinal organoids is modulated by Apc mutation and other colorectal cancer progression factors. Cell. Mol. Life Sci..

[38] Ramanathan S., Douglas S.R., Alexander G.M., Shenoda B.B., Barrett J.E., Aradillas E., Sacan A., Ajit S.K. (2019). Exosome microRNA signatures in patients with complex regional pain syndrome undergoing plasma exchange. J. Transl. Med..

[39] Yeon J.H., Jeong H.E., Seo H., Cho S., Kim K., Na D., Chung S., Park J., Choi N., Kang J.Y. (2018). Cancer-derived exosomes trigger endothelial to mesenchymal transition followed by the induction of cancer-associated fibroblasts. Acta Biomater..

[40] Hakulinen J., Sankkila L., Sugiyama N., Lehti K., Keski-Oja J. (2008). Secretion of active membrane type 1 matrix metalloproteinase (MMP-14) into extracellular space in microvesicular exosomes. J. Cell. Biochem..

[41] Han W., Chen S., Yuan W., Fan Q., Tian J., Wang X., Chen L., Zhang X., Wei W., Liu R. (2016). Oriented collagen fibers direct tumor cell intravasation. Proc. Natl. Acad. Sci. USA.

[42] Fang M., Yuan J., Peng C., Li Y. (2014). Collagen as a double-edged sword in tumor progression. Tumour Biol..

[43] Van Doren S.R. (2015). Matrix metalloproteinase interactions with collagen and elastin. Matrix Biol..

[44] Provenzano P.P., Inman D.R., Eliceiri K.W., Knittel J.G., Yan L., Rueden C.T., White J.G., Keely P.J. (2008). Collagen density promotes mammary tumor initiation and progression. BMC Med..

[45] Conklin M.W., Eickhoff J.C., Riching K.M., Pehlke C.A., Eliceiri K.W., Provenzano P.P., Friedl A., Keely P.J. (2011). Aligned collagen is a prognostic signature for survival in human breast carcinoma. Am. J. Pathol..

[46] Bredfeldt J.S., Liu Y., Conklin M.W., Keely P.J., Mackie T.R., Eliceiri K.W. (2014). Automated quantification of aligned collagen for human breast carcinoma prognosis. J. Pathol. Inform..

[47] Ray A., Slama Z.M., Morford R.K., Madden S.A., Provenzano P.P. (2017). Enhanced Directional Migration of Cancer Stem Cells in 3D Aligned Collagen Matrices. Biophys. J..

[48] Yang N., Mosher R., Seo S., Beebe D., Friedl A. (2011). Syndecan-1 in breast cancer stroma fibroblasts regulates extracellular matrix fiber organization and carcinoma cell motility. Am. J. Pathol..

[49] Wang K., Fei Wu F., Ri Seo B., Fischbach C., Chen W., Hsu L., Gourdon D. (2017). Breast cancer cells alter the dynamics of stromal fibronectin-collagen interactions. Matrix Biol..

[50] Piperigkou Z., Mohr B., Karamanos N., Gotte M. (2016). Shed proteoglycans in tumor stroma. Cell Tissue Res..

[51] Jabłońska-Trypuć A., Matejczyk M., Rosochacki S. (2016). Matrix Metalloproteinases (MMPs), the main extracellular matrix (ECM) enzymes in collagen degradation, as a target for anticancer drugs. J. Enzym. Inhib. Med. Chem..

[52] Malik R., Lelkes P.I., Cukierman E. (2015). Biomechanical and biochemical remodeling of stromal extracellular matrix in cancer. Trends Biotechnol..

[53] Kumar S., Das A., Sen S. (2014). Extracellular matrix density promotes EMT by weakening cell–cell adhesions. Mol. Biosyst..

[54] Hubbard B., Buczek-Thomas J.A., Nugent M.A., Smith M.L. (2015). Fibronectin Fiber Extension Decreases Cell Spreading and Migration. J. Cell. Physiol..

[55] Dupont M., Souriant S., Lugo-Villarino G., Maridonneau-Parini I., Verollet C. (2018). Tunneling nanotubes: Intimate communication between myeloid cells. Front. Immunol..

[56] Sowinski S., Jolly C., Berninghausen O., Purbhoo M.A., Chauveau A., Kohler K., Oddos S., Eissmann P., Brodsky F.M., Hopkins C. (2008). Membrane nanotubes physically connect T cells over long distances presenting a novel route for HIV-1 transmission. Nat. Cell Biol..

[57] Ady J.W., Desir S., Thayanithy V., Vogel R.I., Moreira A.L., Downey R.J., Fong Y., Manova-Todorova K., Moore M.A., Lou E. (2014). Intercellular communication in malignant pleural mesothelioma: Properties of tunneling nanotubes. Front. Physiol..

[58] Guo W., Giancotti F.G. (2004). Integrin signalling during tumour progression. Nat. Rev. Mol. Cell Biol..

[59] Levental K.R., Yu H., Kass L., Lakins J.N., Egeblad M., Erler J.T., Fong S.F., Csiszar K., Giaccia A., Weninger W. (2009). Matrix crosslinking forces tumor progression by enhancing integrin signaling. Cell.

[60] Poltavets V., Kochetkova M., Stuart M., Pitson S.M., Samuel M.S. (2018). The Role of the Extracellular Matrix and Its Molecular and Cellular Regulators in Cancer Cell Plasticity. Front. Oncol..

[61] Koch T.M., Munster S., Bonakdar N., Butler J.P., Fabry B. (2012). 3D Traction Forces in Cancer Cell Invasion. PLoS ONE.

[62] Steinwachs J., Metzner C., Skodzek K., Lang N., Thievessen I., Mark C., Münster S., Aifantis K.E., Fabry B. (2016). Three-dimensional Force Microscopy of Cells in Biopolymer Networks. Nat. Methods..

[63] Samuel M.S., Lopez J.I., Mcghee E.J., Croft D.R., Strachan D., Timpson P., Munro J., Schröder E., Zhou J., Brunton V.G. (2011). Actomyosin-mediated cellular tension drives increased tissue stiffness and beta-catenin activation to induce epidermal hyperplasia and tumor growth. Cancer Cell.

[64] Rilla K., Siiskonen H., Tammi M., Tammi R. (2014). Hyaluronan-coated extracellular vesicles-a novel link between hyaluronan and cancer. Adv. Cancer Res..

[65] Kosaka N. (2016). Decoding the Secret of Cancer by Means of Extracellular Vesicles. J. Clin. Med..

[66] Kolba M.D., Dudka W., Zaręba-Kozioł M., Kominek A., Ronchi P., Turos L., Chroscicki P., Wlodarczyk J., Schwab Y., Klejman A. (2019). Tunneling nanotube-mediated intercellular vesicle and protein transfer in the stroma-provided imatinib resistance in chronic myeloid leukemia cells. Cell Death Dis..

[67] Bölük A., Bilgi C., Kutlu E., Akbaş D., Gökdoğan Edgünlü T. (2020). Tunneling Nanotube-Mediated Mitochondrial Transfer: A New Approach to Cell Protection. Am. J. Biomed. Sci. Res..

[68] Albini A., Iwamoto Y., Kleinman H.K., Martin G.R., Aaronson S.A., Kozlowski J.M., McEwan R.N. (1987). A rapid in vitro assay for quantitating the invasive potential of tumor cells. Cancer Res..

[69] Karamanou K., Franchi M., Vynios D., Brézillon S. (2020). Epithelial-to-mesenchymal Transition and Invadopodia Markers in Breast Cancer: Lumican a Key Regulator. Semin. Cancer Biol..

[70] Haeger A., Krause M., Wolf K., Friedl P. (2014). Cell jamming: Collective invasion of mesenchymal tumor cells imposed by tissue confinement. Biochim. Biophys. Acta Gen. Subj..

[71] Yang Y., Zheng H., Zhan Y., Fan S. (2019). An Emerging Tumor Invasion Mechanism About the Collective Cell Migration. Am. J. Transl. Res..

[72] Heusermann W., Hean J., Trojer D., Steib E., von Bueren S., Graff-Meyer A., Genoud C., Martin K., Pizzato N., Voshol J. (2016). Meisner-Kober, Exosomes surf on filopodia to enter cells at endocytic hot spots, traffic within endosomes, and are targeted to the ER. J. Cell Biol..

[73] Franchi M., Piperigkou Z., Riti E., Masola V., Onisto M., Karamanos N.K. (2020). Long filopodia and tunneling nanotubes define new phenotypes of breast cancer cells in 3D cultures. Matrix Biol. Plus.

[74] Franchi M., Masola V., Bellin G., Onisto M., Karamanos K.A., Piperigkou Z. (2019). Collagen fiber array of peritumoral stroma influences epithelial-to-mesenchymal transition and invasive potential of mammary cancer cells. J. Clin. Med..

[75] Hecht I., Bar-El Y., Balmer F., Natan S., Tsarfaty I., Schweitzer F., Ben-Jacob E. (2015). Tumor Invasion Optimization by Mesenchymal-Amoeboid Heterogeneity. Sci. Rep..

[76] Paszek M.J., Zahir N., Johnson K.R., Lakins J.N., Rozenberg G.I., Gefen A., Reinhart-King C.A., Margulies S.S., Dembo M., Boettiger D. (2005). Tensional homeostasis and the malignant phenotype. Cancer Cell.

[77] Zaman M.H., Trapani L.M., Sieminski A.L., Mackellar D., Gong H., Kamm R.D., Wells A., Lauffenburger D.A., Matsudaira P. (2006). Migration of tumor cells in 3D matrices is governed by matrix stiffness along with cell-matrix adhesion and proteolysis. Proc. Natl. Acad. Sci. USA.

[78] Acerbi I., Cassereau L., Dean I., Shi Q., Au A., Park C., Chen Y.Y., Liphardt J., Hwang E.S., Weaver V.M. (2015). Human breast cancer invasion and aggression correlates with ECM stiffening and immune cell infiltration. Integr. Biol..

[79] Friedl P., Wolf K. (2010). Plasticity of cell migration: A multiscale tuning model. J. Cell Biol..

[80] Friedl P., Alexander S. (2011). Cancer Invasion and the Microenvironment: Plasticity and Reciprocity. Cell.

[81] Fraley S.I., Wu P., He L., Feng Y., Krisnamurthy R., Longmore G.D., Wirtz D. (2015). Three-dimensional matrix fiber alignment modulates cell migration and MT1-MMP utility by spatially and temporally directing protrusions. Sci. Rep..

